# Genomic regions and genes related to inter-population differences in body size in the ground beetle *Carabus japonicus*

**DOI:** 10.1038/s41598-017-08362-7

**Published:** 2017-08-10

**Authors:** Ryohei Komurai, Tomochika Fujisawa, Yutaka Okuzaki, Teiji Sota

**Affiliations:** 1 Department of Zoology, Graduate School of Science, Kyoto University, Sakyo, Kyoto, 606-8502 Japan; 2 Field Science Center for Northern Biosphere, Hokkaido University, Tomakomai, 053-0035 Japan

## Abstract

Body size is a key trait in diversification among animal species, and revealing the gene regions responsible for body size diversification among populations or related species is important in evolutionary biology. We explored the genomic regions associated with body size differences in *Carabus japonicus* ground beetle populations by quantitative trait locus (QTL) mapping of F_2_ hybrids from differently sized parents from two populations using restriction site-associated DNA sequencing and *de novo* assembly of the beetle whole genome. The assembled genome had a total length of 191 Mb with a scaffold N50 of 0.73 Mb; 14,929 protein-coding genes were predicted. Three QTLs on different linkage groups had major effects on the overall size, which is composed chiefly of elytral length. In addition, we found QTLs on autosomal and X chromosomal linkage groups that affected head length and width, thoracic width, and elytral width. We determined the gene loci potentially related to control of body size in scaffolds of the genome sequence, which contained the QTL regions. The genetic basis of body size variation based on a small number of major loci would promote differentiation in body size in response to selection pressures related to variations in environmental conditions and inter-specific interactions.

## Introduction

Body size, a key trait for all organisms, affects various aspects of survival and reproduction of individuals in their interactions with their physical and biological environments. In animals, divergence of body size among related populations is often related to adaptation to differing environmental conditions^1^ and/or mitigation of interactions with other species through resource competition and/or reproductive interference^2–4^. Revealing the genetic basis for body size differences between closely related populations is important in understanding the adaptive divergence of populations, which may ultimately result in speciation.

Genes involved in the control of animal body size have been examined in various mammals and insects^5, 6^. Several pathways have been investigated in *Drosophila melanogaster*, including the insulin signalling pathway, which contributes to the regulation of growth rates and body size in response to environmental cues, such as the presence of nutrients^6^. For example, overexpression of an insulin-like peptide during development can increase body size^7^. Mutations in genes involved in insulin signalling and other developmental pathways regulating larval growth^8^ may also be involved in the evolution of overall body size in insects. However, the genetic basis for body size differences among populations or closely related species remains largely unknown. Although several genetic loci or markers in *Drosophila* show strong associations with geographic variation in body size^9–14^, the relationships between genes or alleles and the regulation of body size that affect body size evolution remain unclear^6^.

**Figure 1:**
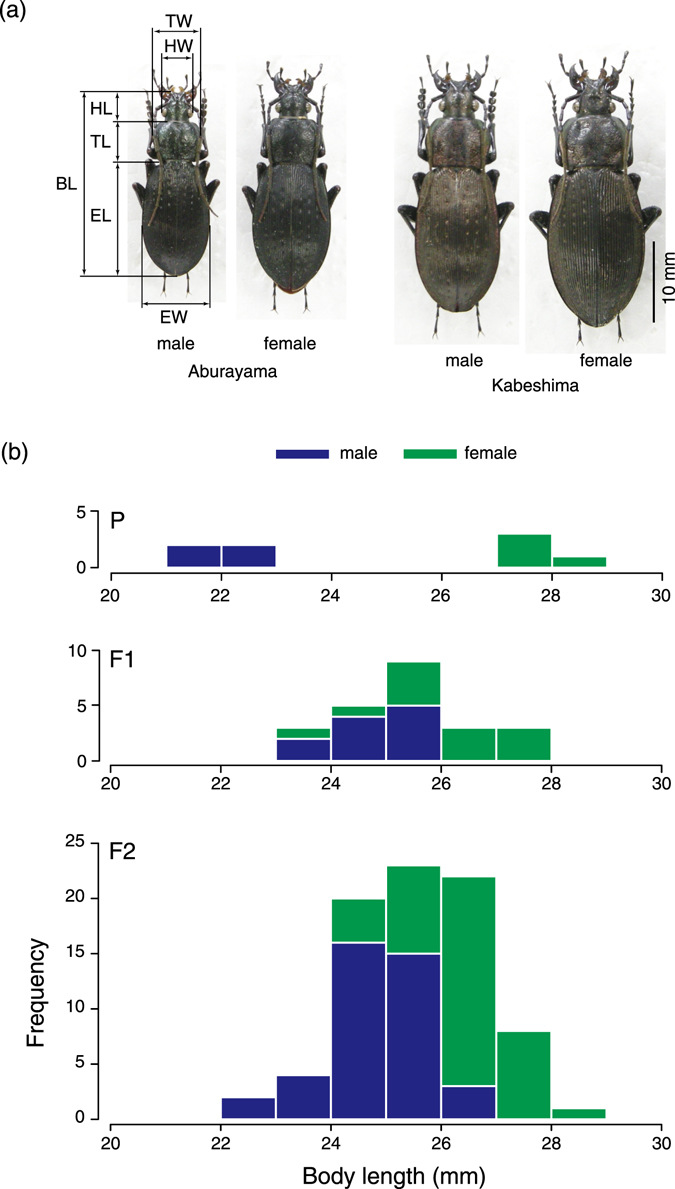
The body lengths of adult P, F_1_, and F_2_ *Carabus japonicus* beetles. (**a**) Males and females from Mt. Aburayama and Kabeshima Island were used in the present study. Measurements of body dimensions are shown. BL, body length; HW, head width; HL, head length; TW, thoracic width; TL, thoracic length; EW, elytral width; EL, elytral length. (**b**) BLs of parents and F_1_ and F_2_ individuals in the crossing experiment.

Here, we explored the genetic basis for inter-population body size differences in the carabid ground beetle *Carabus* (*Ohomopterus*) *japonicus* (Fig. 1a). The subgenus *Ohomopterus*, which is endemic to the Japanese islands, shows marked variation in body size (body length) within and among species^15, 16^. Notably, two or three species with different body sizes co-occur in a large region of the subgenus’ range; the size differences are the main contributors to the avoidance of hybridisation^4^. *C*. *japonicus* is sympatric with one or two larger species existing within the majority of its range^16^; however, it exhibits larger bodies in several solitary islands, consistent with the pattern of character release^17^. Such inter-population divergence might be an initial step in speciation via recurrent secondary contacts between populations of differently sized individuals.

In this study, we used a traditional approach of accessing the loci responsible for quantitative traits (i.e., QTL mapping) combined with whole genome sequencing and gene prediction to determine the genomic region and gene loci responsible for inter-population body size differences. First, we assembled the genomic sequence of *C*. *japonicus* and predicted protein-coding genes on the scaffolds. Second, we obtained F_2_ individuals by crossing females and males from populations with large and small bodies and measured their body size dimensions. Then, we obtained a large number of restriction site-associated DNA (RAD) sequences for F_2_ individuals to construct linkage maps and to perform QTL mapping. The locations of QTLs for body size on the linkage maps were estimated from the correlations between the phenotypic values of a body dimension and genotypes among F_2_ individuals. Finally, we searched for protein-coding genes potentially related to the body size differences by exploring the scaffolds of the *C*. *japonicus* genomic sequence containing QTLs for body size with reference to the known functions of annotated genes.

## Results

**Figure 2:**
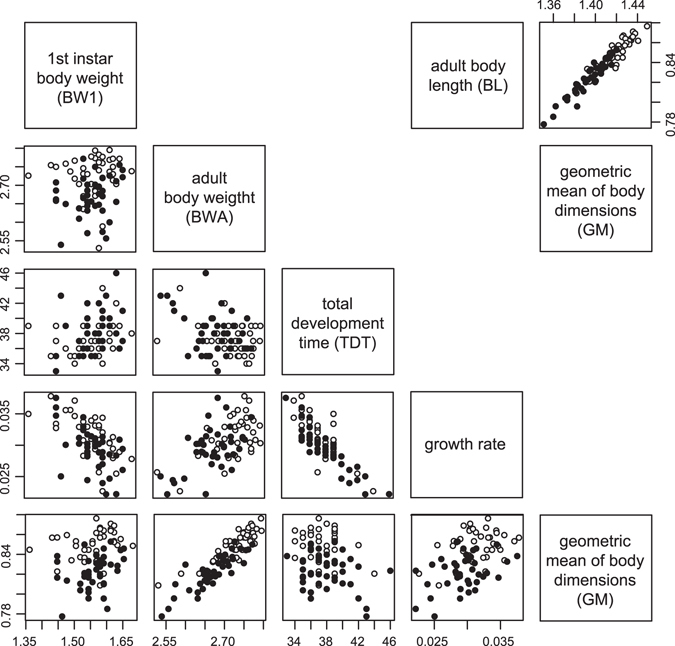
Correlations between developmental trait values and body size dimensions. Open and closed circles indicate male and female individuals, respectively. BW1, BWA and BL are log_10_ transformed values, and GM (geometric mean of all body dimensions) was calculated as the arithmetic mean of all log_10_ transformed body dimensions except BL.

**Figure 3:**
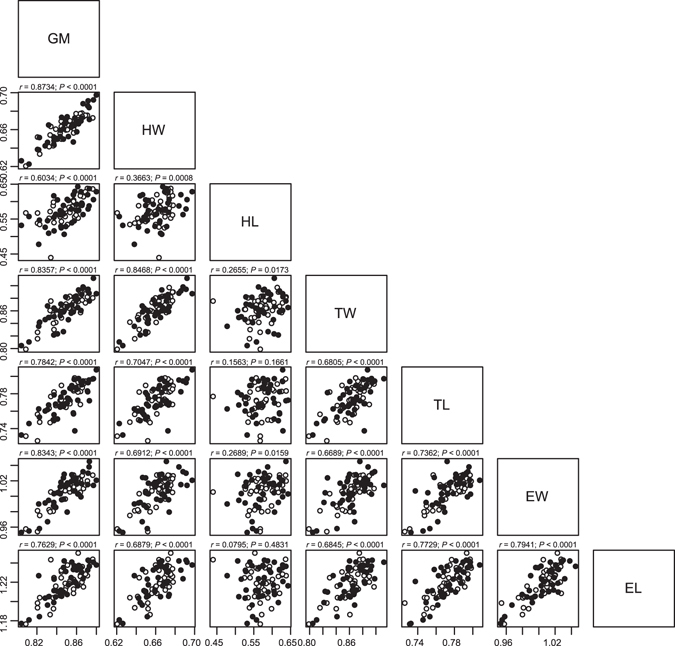
Correlations between body size dimensions. The values in millimetres were log_10_ transformed and adjusted for sex differences. Open and closed circles indicate male and female individuals, respectively. Pearson correlation coefficients (*r*) and *P* values are given in the panels. The body dimensions are described in the legend of Fig. 1.

By experimental crossings, we obtained 40 male and 40 female F_2_ adults (Fig. 1b). Although the initial first instar weight (BW1) and total development time from larval hatching to adult eclosion (TDT) showed no difference between the sexes (BW1, *F*_1,75_ = 0.073, *P* = 0.7879; TDT, *F*_1,77_ = 2.327, *P* = 0.1312), the growth rate in terms of increasing body weight during the immature stages was larger in females than in males (*F*_1,74_ = 7.875, *P* = 0.0064), leading to larger body sizes in females. Excluding the effect of sex, the geometric mean of all adult body dimensions (GM) was positively related to the initial larval body weight (proxy of egg size), developmental time from oviposition to adult emergence, and growth rate (Fig. 2; Table 1). Of these three variables, growth rate had the largest effect, while the remaining two variables had comparable effects. Overall body length (BL; Fig. 1a) was closely correlated with GM (*r* = 0.962, *P* < 0.0001) and showed almost identical responses to the four variables (results not shown). Because of the sexual dimorphism in body dimensions, we used sex-adjusted dimensions in the QTL analyses. Hereafter, symbols for individual body dimensions (HL, HW, TL, TW, EL and EW; Fig. 1a) and overall body size dimensions (GM and BL) are used for log_10_ transformed, sex-adjusted values. All of these variables were significantly correlated with each other except for two cases (Fig. 3).

**Table 1:** Effects of sex, developmental time (DT), larval weight at hatching, and growth rate on the geometric mean of all adult body dimensions (GM; log10 transformed).

Variable	Effect	SE	df	*t*	*P*
Sex [female-male]	0.419	0.063	1,70.11	6.67	<0.0001
Initial larval weight (BW1)	0.613	0.072	1,70.25	8.49	<0.0001
Development time (TDT)	0.187	0.069	1,70.11	2.73	0.0080
Growth rate	0.824	0.088	1,70.17	9.37	<0.0001

All variables (other than sex) were standardised. Family was incorporated as a random variable.

Sequence assembly resulted in the construction of 76,540 scaffolds with an N50 of 734,069 bp (max. 6,537,065) and total length of 191,032,029 bp (N, 8%; GC, 35.0%). A total of 14,929 protein-coding genes were predicted; of these, 10,624 (71%) were assigned to known genes by a blast search.

**Figure 4:**
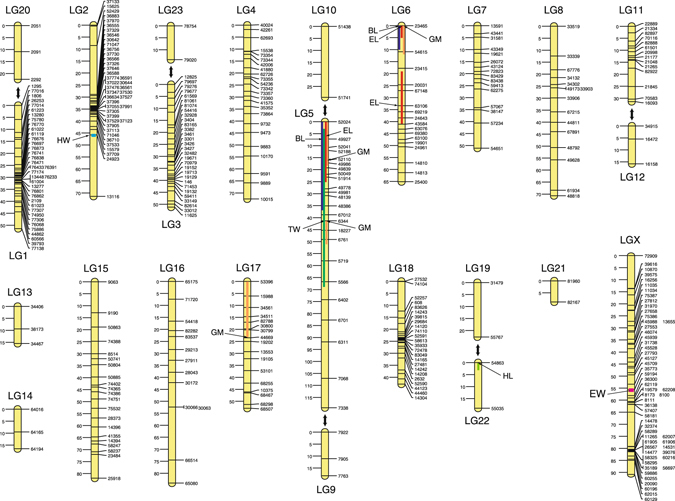
Linkage map of *Carabus japonicus* showing the locations (peak and range) of the body dimension quantitative trait loci (QTLs). Two-way arrows connect linkage groups probably located on the same chromosomes.

We constructed 23 autosomal linkage groups with a total length of 1,029 cM from 319 RAD loci and an X-chromosome linkage group of 91 cM from 56 RAD loci (a total of 1,120 cM with 375 markers; Fig. 4). Based on shared scaffolds, the number of autosomal linkage groups could be reduced to 17, which was still larger than the actual haploid autosome number of 13.

**Figure 5:**
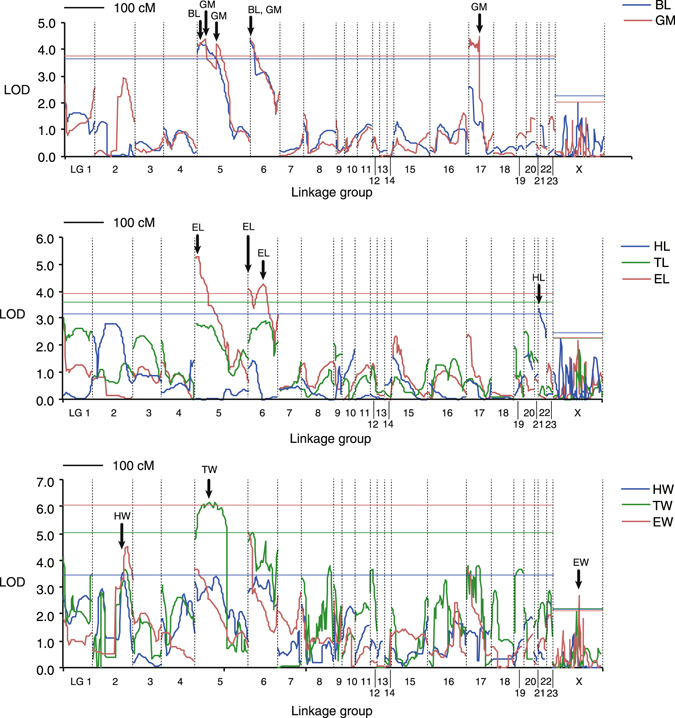
LOD scores for QTLs of body dimensions in the composite interval mapping analysis. Horizontal lines indicate threshold LOD values at α = 0.05. Arrows indicate peaks of LOD score.

Among the autosomal linkage groups, we found two QTLs of large effect for body length (BL) and geometric mean of all body dimensions (GM) on LG5 and LG6 (*R* ^2^ = 0.26–0.35; Figs 4 and 5; Table 2). Of the components of BL, elytral length (EL), which comprises 63.1% of BL on average, had two QTLs corresponding to those of BL on LG5 and LG6 and an additional QTL on LG6; these QTLs had large effects (*R* ^2^ = 0.36−0.44). In addition, a QTL for GM occurred on LG17 (*R* ^2^ = 0.43) and was related to peaks of other dimensions, which were not significant. We found one QTL for head width (HW) on LG2, one QTL for head length (HL) on LG22, and one QTL for thoracic width (TW) on LG5, but no QTL for thoracic length (TL). All QTLs, except that for HW, exhibited large dominance effects (Table 2). The X chromosome contained a QTL for elytral width (EW), but it had a relatively low logarithm of odds (LOD) score (Table 2; Fig. 5).

**Table 2:** Significant QTLs for body size dimensions.

Trait	LG	Position (cM)		LOD	Effect		*R* ^2^
		Peak	Range	peak value	*a*	*d*	
BL	LG5	7.3	0.0–37.1	4.283	0.0111	0.0216	0.2862
BL	LG6	0.0	0.0–10.0	4.299	0.0103	0.0191	0.3514
GM	LG5	16.6	0.0–18.3	4.390	0.0187	0.0212	0.2733
GM	LG5	41.3	41.3–51.1	4.216	0.0216	0.0199	0.2620
GM	LG6	0.0	0.0–11.9	4.377	0.0187	0.0208	0.3546
GM	LG17	.23.2	0.0–23.2	4.466	-0.0226	0.0176	0.4304
HL	LG22	0.0	0.0–3.0	3.350	0.0437	0.0246	0.3355
HW	LG2	45.8	45.5–46.8	3.567	-0.0291	0.0049	0.4790
TW	LG5	45.3	3.0–68.7	6.056	0.0297	0.0345	0.3078
EL	LG5	6.0	0.0–25.1	5.295	0.0098	0.0261	0.4397
EL	LG6	0.0	0.0–5.0	4.119	0.0125	0.0256	0.4101
EL	LG6	33.5	19.8–41.1	4.274	0.0079	0.0341	0.3606
EW	LGX	55.6	55.5–56.6	2.655	-0.0118	—	0.2373

Trait: body size dimensions, which were log10-transformed and sex-adjusted.

LG: linkage group.

Effect: *a*, additive; *d*, dominance. *R* ^2^, proportion of variance explained.

**Figure 6:**
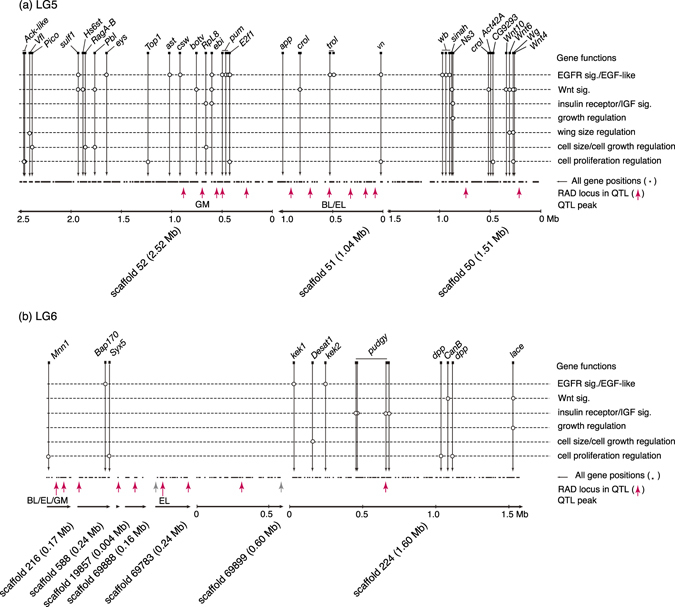
Genes potentially related to body size QTLs on scaffolds of two linkage groups, LG5 (**a**) and LG6 (**b**). Red upward asterisks indicate restriction site-associated DNA (RAD) loci with significant QTL LOD scores. Genes shown here are those related to the regulation of cell proliferation, cell size/cell growth, and wing size, and growth; and those involved in or regulating the insulin receptor/insulin-like growth factor signalling pathway, the Wnt signalling pathway, and the epidermal growth factor/epidermal growth factor receptor signalling pathway. Open circles indicate functions known for the gene located at the position. See Table S3 (Supplementary Information) for precise gene names and gene ontology terms.

From 2,304 gene loci involved in 24 scaffolds with body size QTLs, we obtained 1,566 RefSeq protein IDs and determined 110 candidate genes by referring to the gene ontology terms (Table 3; see also Table S3, Supplementary Information, for details of all candidate genes found in the scaffolds with QTL regions). In the two linkage groups (LG5, LG6) with major QTLs, LG5 contained *Wnt*/*wingless* genes and several other genes related to the regulation of cell proliferation, cell size, and wing size; namely, the *vein* locus existed near the peak position of the QTL for BL/EL, and the *ribosomal protein L8*, *E2F transcription factor 1* and *topoisomerase 1* loci occurred around the peak position of the QTL for GM (Fig. 6a; Table S3, Supplementary Information). The beetle elytra correspond to the forewings of flies; therefore, the *wingless* gene may be related to the regulation of elytral size. The peak position of the QTL for EL is located in the *terribly reduced optic lobes* (*trol*) gene region; however, whether this gene is related to the regulation of body size is unknown. The QTL region for HW/GM on LG5 (not shown in Fig. 6a) contained the *CG11940* (*pico*), *humpty dumpty*, *CG4207* (*bonsai*) and *Stat92E* loci, which are involved in regulation of cell proliferation/size and growth (Table S3, Supplementary Information). For LG6 (Fig. 6b), the composition and order of scaffolds are unclear. Near the QTL for BL/EL/GM on LG6, we found the *menin 1* and *syntaxin 5* genes, which are involved in regulation of cell proliferation. The QTL region for EL in LG6 contains the *pudgy* loci; this gene negatively regulates the insulin receptor signalling pathway. Additionally, the *decapentaplegic* gene, a key morphogen affecting cell proliferation, exists near the EL QTL region. For the QTL for HW on LG2, we found the *dumpy* and *Keren* genes, which are involved in regulation of wing size and cell proliferation, respectively (Table S3, Supplementary Information). For the QTL for HL on LG22, we found the *Smurf* gene, involved in cell size regulation, and the *frizzled 2* gene of the Wnt signalling pathway. Also, the *CG7467* (*osa*) gene, which affects the Wnt signalling pathway, was found in the QTL for EW (Table S3, Supplementary Information).

**Table 3:** Scaffolds of *C*. *japonicus* with body size QTLs.

Linkage group	Scaffold no.	QTL traits	Length, bp	No. gene loci	No. candidate genes
LG2	226	HW*	51101	0	0
LG2	350	HW	4247482	325	12
LG5	50	BL, TW	1511372	122	10
LG5	51	BL*, GM, TW, EL*	1040234	68	4
LG5	52	BL, GM*, TW, EL	2524599	292	17
LG5	119	GM*, TW	6535796	603	19
LG5	1761	GM, TW*	1462	6	0
LG5	69886	TW	348051	12	0
LG6	216	BL*, GM*, EL*	167963	15	1
LG6	588	GM	226143	30	2
LG6	19857	EL	4139	1	0
LG6	69888	EL	158683	8	0
LG6	69783	EL*	236463	23	0
LG6	69899	EL	599710	51	0
LG6	224	EL	1599484	158	7
LG6	4437	EL	11584	0	0
LG17	15	GM	341150	27	0
LG17	54	GM	1309841	101	3
LG17	2833	GM*	75834	11	0
LG17	3109	GM	41374	5	0
LG17	3143	GM	34205	1	0
LG22	59	HL*	3237164	265	21
LGX	341	EW	799159	76	5
LGX	644	EW	874966	104	9
Total			2577959	2304	110

Asterisks indicate that the LOD peaks of the traits exist on the corresponding scaffolds.

For candidate gene numbers, the numbers accounting for multi-locus genes on the same scaffold are given.

## Discussion

The inferred genome size of *C*. *japonicus* is approximately 191 Mb, and the number of predicted genes is ~14,900. This moderate genome size and gene number may render this species suitable for the study of the genomic basis of adaptive traits. However, a disadvantage of this species is its low fecundity, which makes construction of a large F_2_ population difficult. In this study, the use of multiple parents was necessary to obtain F_1_ beetles and a total of 80 F_2_ beetles with a sex ratio of 1:1. The limited number of F_2_ individuals was a factor reducing the accuracy of the QTL analysis.

Through the hybridisation of differently sized males and females from two populations, we obtained F_2_ individuals showing large variation in body size (Fig. 1b). Thus, the body size dimensions likely had a polygenic basis. In F_2_ individuals, larger body sizes were related positively to larger live body weight of first instars and larger growth rate in body weight from the first instar to the emerging adult stage (Fig. 2). Therefore, the larger initial size (egg size) and the larger immature growth rate contributed to the larger adult body size^17^.

We found that two regions on LG5 and LG6 regulated the overall body size (BL and GM), with relatively large effects. These regions contained QTLs for EL, which occupies a large proportion of BL, and a QTL for TW. There was another QTL for GM on LG17, where we found no QTL for body part dimensions, suggesting that this QTL regulates sizes of body parts in a different way from that of the QTLs on LG5 and LG6. If the body size of *C*. *japonicus* is actually controlled by a few gene loci with large phenotypic effects, body size divergence may occur readily in response to different habitat conditions, such as temperature and food resources^17^.

The pattern of body size QTL distribution is similar to that found in previous QTL studies for *D*. *melanogaster* and its allied species, in which a major QTL region for wing and other body sizes was located on chromosome 3 and an additional QTL region was located on chromosome 2^9, 11, 14^. The *Drosophila* studies focused on latitudinal variations in body size, and adaptive body size divergence between beetles and flies may have a similar genetic basis. In *Drosophila* species, several genes with allelic differences are associated with latitudinal body size variations. However, the functions of those genes in controlling body size remain undetermined. In this *C*. *japonicus* study, determination of responsible genes was difficult due to the low marker density and limited linkage map. The QTL regions may involve some degree of sequence differences in several regulatory genes that affect cell proliferation, cell size, or growth rate. Future studies are required to detect the sequence differences in the QTL regions that correspond to body size differences by re-sequencing multiple samples from populations with different body sizes.

## Methods

### Experimental crossing and body size analysis

We constructed an F_2_ adult population from four pairs of large females from Kabeshima Island, Saga (33°32′55″N, 129°52′56″E, 30 m a.s.l.), and small males from Mt. Aburayama, Fukuoka (33°30′43″N, 130°21′52″E, 580 m a.s.l.; Fig. 1a). By crossing F_1_ males and females, we obtained 40 adult males and 40 adult females in the F_2_ generation for linkage map construction and QTL analysis (Fig. 1b). The rearing methods were identical to those described by Okuzaki *et al*.^17^.

We measured the body weights of the first, second, and third instar and adult individuals just after hatching/eclosion. We also measured the duration of the various developmental stages to analyse the variations in growth and development patterns in F_2_ individuals. Using log_10_ transformed values for the initial first instar weight (BW1) and adult body weight at eclosion (BWA), and the total development time from hatching to adult eclosion (TDT; not log_10_ transformed), we calculated the larval growth rate as (BWA–BW1)/TDT. Approximately 1 month after adult emergence, F_2_ beetles were fixed in ethanol. We measured six body dimensions (Fig. 1a; Table S1, Supplementary Information) using a digital calliper (Mitsutoyo, Japan): the BL, from the apex of the labrum to the apex of the elytra; the HW, represented by the distance between the outer margins of the eyes; the TW, the maximum width of the thorax; the TL, the length of the thorax; the EW, the maximum width of the elytra; and the EL, from the base to the tip of the right elytra. HL was determined as BL–TL–EL. All body size dimensions were log_10_ transformed. Although BL is the most straightforward measurement for overall body size and has been used in previous studies of *Carabus* beetles^4, 15, 16^, we also used log_10_ transformed value of the geometric mean of all body dimensions except BL^18–20^. The logged geometric mean is effectively the arithmetic mean of log_10_ transformed dimensions and is denoted simply by GM in this paper. Consistent with sexual dimorphism in body size, the females were generally larger than males. Therefore, male body dimensions were adjusted so that their means were equal to the means of females.

### RAD sequences

Using the Genomic DNA Purification Kit (Promega) or DNeasy Kit (Qiagen), total DNA was extracted from the gonads and muscle tissues of the parents and F_2_ adults preserved in ethanol. The library for RAD sequencing was constructed as described by Etter *et al*.^21^. The extracted DNA was adjusted to 25 ng/μl, and a 12-μl aliquot was digested with the endonuclease *Pst*I; the digested fragments were ligated with P1 adaptors containing a 5-bp barcode sequence. Subsequent library construction procedures and 100-bp single-end sequencing with Illumina HiSeq. 2000 were conducted by Hokkaido System Science Co. Ltd. (Sapporo, Japan). The RAD sequences obtained and their accession numbers in the DNA Data Bank of Japan are listed in Table S2 (Supplementary Information).

### Genome sequencing and gene prediction

Total DNA was extracted from the testes of two males from Kabeshima Island. A paired-end library with 170-bp insertion (j-1–170) and two mate-pair libraries with 2,000-bp and 5,000-bp insertions (j-1-2K, j-2-5K) were constructed and sequenced using one lane of a HiSeq. 4000 sequencer (Illumina). We obtained 297 M clean reads for j-1-170 (Q20, 98.6%), 117 M reads for j-1-2K (Q20, 98.3%) and 110 M reads for j-2-5K (Q20, 98.0%). The raw reads were deposited at the DNA Data Bank of Japan (DDBJ DRA; BioProject, PRJDB5403; DRR089090-089092). The sequences were assembled using Platanus version 1.2.1^22^. We annotated the assembled scaffolds by combining evidence from multiple gene prediction procedures. Ab-initio gene prediction was conducted with Augustus version 3.1^23^ and GeneMark-ES version 4.32^24^. Augustus was run with parameters trained using *Tribolium castaneum*, which is the closest available genome. A self-training procedure implemented in GeneMark-ES was used to optimise the parameters for prediction. In addition to the ab-initio predictions, the coding sequences (CDS) of 15,607 genes predicted in the *Carabus uenoi* draft genome (T. Fujisawa *et al*., unpublished) were aligned to the scaffolds using Exonerate^25^ (Slater & Birney 2005) with a percentage similarity of 80%. The evidence from ab-initio prediction and CDS alignment was combined by EVidenceModeler^26^, with evidence weights of 5 for CDS alignment and 1 for ab-initio prediction. The translated sequences of the predicted gene set were searched against the RefSeq protein database and its *D*. *melanogaster* subset using BLASTp (BLAST + ver. 2.2.30)^27^ to obtain orthological information.

### Linkage map construction

We used the genome sequence of *C*. *japonicus* to determine the RAD loci segregating between parents and genotyping of F_2_ individuals for the orthologous loci using the Stacks programme (ver. 1.35)^28^. The karyotype of the genus *Carabus* is 2n = 28, with an XY sex chromosome system^29^. After genotyping, we discriminated loci on the X chromosomes from those on autosomes in males, as the male X chromosome markers would have contained no heterozygote. We constructed autosomal linkage maps and an X chromosomal linkage map separately using the JoinMap^®^ 4.1 programme (Kyazma V.B., Wageningen, The Netherlands). For autosomal chromosomes, we used 1,463 loci and obtained 23 linkage groups with a total length of 1,029 cM from 319 loci at an LOD score of 6. For the X chromosome, we used 171 putative X-chromosome loci from males (haploid) and obtained a linkage map with 56 loci and 90.83 cM in length at an LOD score of 2.

### QTL mapping

We conducted composite interval mapping using Windows QTL Cartographer version 2.5_011^30^ for autosomal loci with male and female data combined (F_2_ cross) and for the X chromosome with male data only because the linkage map was constructed exclusively from male data. We conducted composite interval mapping^31^ with a walk speed of 1 cM (using a standard background control method with forward and backward regression, control marker number of 5, and probability of into/out of a 0.1 window size of 10 cM). The LOD threshold values at α = 0.05 were obtained by 1,000 permutations.

### Candidate genes

For the predicted genes located in the QTL regions, functional annotations were made using DAVID Bioinformatics Resources 6.8^32, 33^. The RefSeq protein ID for each locus based on *Drosophila melanogaster* genes was used for input, and three gene ontology terms (biological process, cell component, and molecular function), protein domains (InterPro), and pathway (KEGG) annotations were obtained. *D*. *melanogaster* was used as the background genome in the DAVID annotation. We used genes existing in scaffolds with the QTLs of body dimensions. We focused on genes related functionally to the regulation of cell proliferation, cell size, cell growth, wing size, and general growth. In addition, we considered genes involved in pathways related to the regulation of body size and growth, the Wnt signalling pathway, the insulin receptor signalling pathway, the insulin-like growth factor signalling pathway, and the epidermal growth factor/epidermal growth factor receptor signalling pathway^6, 8^.

## Electronic supplementary material

Supplementary Information
