# Supplementary material for: Genomic regions and genes related to inter-population differences in body size in the ground beetle Carabus japonicus
Source: Sci Rep. 2017 Aug 10;7:7773. doi: 10.1038/s41598-017-08362-7 (PMC5552851; doi:10.1038/s41598-017-08362-7)
Supplement: Supplementary file 1 — Supplementary Information [file 41598_2017_8362_MOESM1_ESM.pdf]

Supplementary Information

Genomic regions and genes related to inter-population differences in body size in the ground beetle *Carabus japonicus*

Ryohei Komurai[1], Tomochika Fujisawa[1], Yutaka Okuzaki[2] & Teiji Sota[1]*

[1]Department of Zoology, Graduate School of Science, Kyoto University, Sakyo, Kyoto 606-8502, Japan. [2]Field Science Center for Northern Biosphere, Hokkaido University, Kita, Sapporo, 060-0811, Japan.

Table S1. Body dimensions of F2 individuals. (a) Original $\log_{10}$-transformed values.
(b) Values of male dimensions adjusted for sexual differences are given.

(a) Original data

ID	sex	BL	HW	HL	TW	TL	EW	EL	GM
F07_2	F	1.4199	0.6431	0.5399	0.8302	0.7820	1.0033	1.2247	0.8372
F07_37	F	1.4244	0.6640	0.5860	0.8641	0.7870	1.0117	1.2199	0.8555
F07_45	F	1.4259	0.6497	0.5431	0.8488	0.7896	1.0063	1.2307	0.8447
F07_60	F	1.4340	0.6721	0.5748	0.8774	0.7877	1.0235	1.2374	0.8622
F07_65	F	1.3940	0.6385	0.5383	0.8156	0.7564	0.9812	1.1935	0.8206
F07_69	F	1.3820	0.6205	0.5676	0.7989	0.7313	0.9576	1.1766	0.8088
F07_9	F	1.4341	0.6709	0.6256	0.8573	0.7827	1.0202	1.2275	0.8640
F08_41	F	1.4219	0.6564	0.6044	0.8733	0.7834	1.0119	1.2128	0.8570
F08_42	F	1.4198	0.6650	0.5825	0.8679	0.7718	1.0144	1.2189	0.8534
F08_44	F	1.4320	0.6739	0.5806	0.8760	0.7820	1.0208	1.235	0.8614
F08_59	F	1.4138	0.6596	0.5717	0.8708	0.7644	1.0148	1.2145	0.8493
F08_62	F	1.4145	0.6699	0.5785	0.8645	0.7704	0.9964	1.2119	0.8486
F08_63	F	1.4357	0.6755	0.6378	0.8821	0.7832	1.0198	1.2268	0.8709
F08_71	F	1.4157	0.6612	0.6199	0.8671	0.7569	1.0275	1.2085	0.8569
F08_74	F	1.4266	0.6739	0.6137	0.8971	0.7912	1.01	1.2152	0.8669
F08_76	F	1.4175	0.6668	0.5706	0.8831	0.7686	1.0111	1.2191	0.8532
F13_1	F	1.3981	0.6335	0.5345	0.8420	0.7474	0.9751	1.204	0.8228
F13_12	F	1.4140	0.6605	0.5637	0.8416	0.7664	0.9856	1.2159	0.8390
F13_13	F	1.4255	0.6779	0.5893	0.8899	0.7889	1.0264	1.2202	0.8654
F13_17	F	1.4226	0.6696	0.5688	0.8681	0.7863	1.0056	1.2212	0.8533
F13_18	F	1.3945	0.6519	0.5673	0.8259	0.7259	0.9542	1.1984	0.8206
F13_19	F	1.4191	0.6634	0.4395	0.8756	0.7769	1.0055	1.2434	0.8340
F13_23	F	1.4097	0.6561	0.5568	0.8595	0.7723	1.0069	1.2085	0.8434
F13_24	F	1.4161	0.6696	0.5572	0.8667	0.7716	1.0072	1.2188	0.8485
F13_3	F	1.4406	0.6755	0.5529	0.8749	0.7933	1.0228	1.2503	0.8616
F13_30	F	1.4063	0.6425	0.5882	0.8535	0.7500	0.9806	1.2038	0.8364
F13_32	F	1.4007	0.6522	0.5982	0.8401	0.7495	0.9875	1.1925	0.8367
F13_36	F	1.4285	0.6773	0.6168	0.8899	0.7677	1.005	1.226	0.8638
F13_38	F	1.4377	0.6755	0.6280	0.8727	0.7945	1.0145	1.2284	0.8689
F13_39	F	1.4356	0.6742	0.5927	0.8772	0.7870	1.0268	1.2362	0.8657
F13_4	F	1.4160	0.6690	0.5249	0.8623	0.7738	1.0127	1.2246	0.8445
F13_40	F	1.4202	0.6643	0.5835	0.8643	0.7735	1.0099	1.2187	0.8524
F13_46	F	1.4491	0.6727	0.6363	0.8806	0.7970	1.0273	1.2438	0.8763

F13_48	F	1.4322	0.6696	0.5565	0.8694	0.7982	1.0195	1.2347	0.8580
F13_5	F	1.4294	0.6773	0.5615	0.8760	0.7853	1.0228	1.2339	0.8595
F13_53	F	1.4049	0.6415	0.5523	0.8494	0.7632	0.9824	1.2052	0.8323
F13_6	F	1.4260	0.6599	0.5343	0.8806	0.7750	1.0172	1.2378	0.8508
F13_67	F	1.4280	0.6650	0.6007	0.8607	0.7851	1.0069	1.2229	0.8569
F13_7	F	1.4211	0.6634	0.5272	0.8623	0.7728	1.0093	1.2324	0.8446
F13_81	F	1.3922	0.6542	0.5715	0.8607	0.7469	0.9799	1.1864	0.8333
M07_10	M	1.3907	0.6215	0.5745	0.7980	0.7551	0.9893	1.1802	0.8198
M07_27	M	1.4153	0.6411	0.6137	0.8317	0.7679	0.9647	1.2055	0.8374
M07_50	M	1.3996	0.6345	0.5647	0.8274	0.7597	0.9844	1.1952	0.8277
M07_66	M	1.3810	0.6160	0.6013	0.7926	0.7229	0.9574	1.1693	0.8099
M07_73	M	1.3960	0.6243	0.5888	0.8169	0.7484	0.9824	1.1877	0.8247
M08_43	M	1.4053	0.6362	0.5326	0.8527	0.7704	0.9866	1.2075	0.8310
M08_47	M	1.4012	0.6392	0.6274	0.8363	0.7464	0.9685	1.1867	0.8341
M08_52	M	1.4064	0.6294	0.5786	0.8488	0.7589	0.9814	1.2031	0.8334
M08_57	M	1.4049	0.6184	0.6052	0.8293	0.7319	0.9897	1.2036	0.8297
M08_58	M	1.4127	0.6631	0.5657	0.8653	0.7667	0.9953	1.2133	0.8449
M08_61	M	1.4053	0.6358	0.5268	0.8407	0.7632	0.9934	1.2113	0.8285
M08_70	M	1.3930	0.6431	0.5167	0.8533	0.7425	0.9782	1.2015	0.8226
M08_72	M	1.4127	0.6428	0.6223	0.8467	0.7459	1.0106	1.2069	0.8459
M08_75	M	1.3891	0.6457	0.5916	0.8378	0.7432	0.945	1.1777	0.8235
M08_77	M	1.3942	0.6444	0.6141	0.8535	0.7112	0.972384	1.191185	0.8311
M08_79	M	1.4141	0.6558	0.5744	0.8591	0.7696	1.0035	1.2125	0.8458
M13_11	M	1.3827	0.6243	0.5604	0.8299	0.7398	0.9686	1.1764	0.8166
M13_14	M	1.3807	0.6226	0.5492	0.8323	0.7419	0.951	1.1751	0.8120
M13_15	M	1.4161	0.6640	0.5912	0.8829	0.7711	0.9795	1.2112	0.8500
M13_16	M	1.4003	0.6382	0.4956	0.8312	0.7554	0.994	1.2125	0.8212
M13_20	M	1.3825	0.6311	0.5169	0.8418	0.7500	0.9836	1.1823	0.8176
M13_21	M	1.3714	0.6180	0.5313	0.8388	0.7356	0.9325	1.1667	0.8038
M13_22	M	1.3987	0.6599	0.5497	0.8690	0.7684	0.9972	1.194	0.8397
M13_26	M	1.3957	0.6415	0.5609	0.8545	0.7609	0.9889	1.1894	0.8327
M13_28	M	1.3997	0.6328	0.5510	0.8293	0.7393	0.9617	1.2057	0.8200
M13_29	M	1.3733	0.6368	0.4967	0.8204	0.7409	0.9602	1.1754	0.8051
M13_31	M	1.3867	0.6215	0.5897	0.7945	0.7248	0.97	1.1809	0.8136
M13_33	M	1.3810	0.6198	0.5418	0.8355	0.7297	0.9539	1.1817	0.8104
M13_34	M	1.3509	0.5981	0.5185	0.7757	0.7064	0.9186	1.1476	0.7775
M13_35	M	1.3731	0.6142	0.5140	0.8171	0.7340	0.9568	1.1739	0.8017
M13_49	M	1.4098	0.6577	0.6053	0.8627	0.7561	0.9806	1.203	0.8442

M13_51	M	1.3833	0.6311	0.5488	0.8233	0.7385	0.9777	1.1806	0.8167
M13_54	M	1.3818	0.6451	0.5548	0.8299	0.7409	0.9609	1.1759	0.8179
M13_55	M	1.4201	0.6693	0.6139	0.8583	0.7813	0.9862	1.2083	0.8529
M13_56	M	1.3886	0.6232	0.5043	0.8217	0.7526	0.9689	1.1936	0.8107
M13_64	M	1.4094	0.6382	0.5626	0.8643	0.7713	0.9682	1.2071	0.8353
M13_68	M	1.4066	0.6444	0.5819	0.8569	0.7642	0.9817	1.2007	0.8383
M13_78	M	1.3599	0.5944	0.5431	0.7815	0.7196	0.9205	1.1513	0.7851
M13_8	M	1.3826	0.6099	0.4676	0.8064	0.7364	0.9555	1.1972	0.7955
M13_80	M	1.3622	0.6232	0.5331	0.8122	0.7270	0.9236	1.1547	0.7956

(b) Values of male dimensions adjusted for sexual differences.

ID	sex	BL	HW	HL	TW	TL	EW	EL	GM
F07_2	F	1.4199	0.6431	0.5399	0.8302	0.7820	1.0033	1.2247	0.8372
F07_37	F	1.4244	0.6640	0.5860	0.8641	0.7870	1.0117	1.2199	0.8555
F07_45	F	1.4259	0.6497	0.5431	0.8488	0.7896	1.0063	1.2307	0.8447
F07_60	F	1.4340	0.6721	0.5748	0.8774	0.7877	1.0235	1.2374	0.8622
F07_65	F	1.3940	0.6385	0.5383	0.8156	0.7564	0.9812	1.1935	0.8206
F07_69	F	1.3820	0.6205	0.5676	0.7989	0.7313	0.9576	1.1766	0.8088
F07_9	F	1.4341	0.6709	0.6256	0.8573	0.7827	1.0202	1.2275	0.8640
F08_41	F	1.4219	0.6564	0.6044	0.8733	0.7834	1.0119	1.2128	0.8570
F08_42	F	1.4198	0.6650	0.5825	0.8679	0.7718	1.0144	1.2189	0.8534
F08_44	F	1.4320	0.6739	0.5806	0.8760	0.7820	1.0208	1.2350	0.8614
F08_59	F	1.4138	0.6596	0.5717	0.8708	0.7644	1.0148	1.2145	0.8493
F08_62	F	1.4145	0.6699	0.5785	0.8645	0.7704	0.9964	1.2119	0.8486
F08_63	F	1.4357	0.6755	0.6378	0.8821	0.7832	1.0198	1.2268	0.8709
F08_71	F	1.4157	0.6612	0.6199	0.8671	0.7569	1.0275	1.2085	0.8569
F08_74	F	1.4266	0.6739	0.6137	0.8971	0.7912	1.0100	1.2152	0.8669
F08_76	F	1.4175	0.6668	0.5706	0.8831	0.7686	1.0111	1.2191	0.8532
F13_1	F	1.3981	0.6335	0.5345	0.8420	0.7474	0.9751	1.2040	0.8228
F13_12	F	1.4140	0.6605	0.5637	0.8416	0.7664	0.9856	1.2159	0.8390
F13_13	F	1.4255	0.6779	0.5893	0.8899	0.7889	1.0264	1.2202	0.8654
F13_17	F	1.4226	0.6696	0.5688	0.8681	0.7863	1.0056	1.2212	0.8533
F13_18	F	1.3945	0.6519	0.5673	0.8259	0.7259	0.9542	1.1984	0.8206
F13_19	F	1.4191	0.6634	0.4395	0.8756	0.7769	1.0055	1.2434	0.8340
F13_23	F	1.4097	0.6561	0.5568	0.8595	0.7723	1.0069	1.2085	0.8434
F13_24	F	1.4161	0.6696	0.5572	0.8667	0.7716	1.0072	1.2188	0.8485
F13_3	F	1.4406	0.6755	0.5529	0.8749	0.7933	1.0228	1.2503	0.8616
F13_30	F	1.4063	0.6425	0.5882	0.8535	0.7500	0.9806	1.2038	0.8364

F13_32	F	1.4007	0.6522	0.5982	0.8401	0.7495	0.9875	1.1925	0.8367
F13_36	F	1.4285	0.6773	0.6168	0.8899	0.7677	1.0050	1.2260	0.8638
F13_38	F	1.4377	0.6755	0.6280	0.8727	0.7945	1.0145	1.2284	0.8689
F13_39	F	1.4356	0.6742	0.5927	0.8772	0.7870	1.0268	1.2362	0.8657
F13_4	F	1.4160	0.6690	0.5249	0.8623	0.7738	1.0127	1.2246	0.8445
F13_40	F	1.4202	0.6643	0.5835	0.8643	0.7735	1.0099	1.2187	0.8524
F13_46	F	1.4491	0.6727	0.6363	0.8806	0.7970	1.0273	1.2438	0.8763
F13_48	F	1.4322	0.6696	0.5565	0.8694	0.7982	1.0195	1.2347	0.8580
F13_5	F	1.4294	0.6773	0.5615	0.8760	0.7853	1.0228	1.2339	0.8595
F13_53	F	1.4049	0.6415	0.5523	0.8494	0.7632	0.9824	1.2052	0.8323
F13_6	F	1.4260	0.6599	0.5343	0.8806	0.7750	1.0172	1.2378	0.8508
F13_67	F	1.4280	0.6650	0.6007	0.8607	0.7851	1.0069	1.2229	0.8569
F13_7	F	1.4211	0.6634	0.5272	0.8623	0.7728	1.0093	1.2324	0.8446
F13_81	F	1.3922	0.6542	0.5715	0.8607	0.7469	0.9799	1.1864	0.8333
M07_10	M	1.4172	0.6497	0.5888	0.8269	0.7813	1.0238	1.2098	0.8467
M07_27	M	1.4418	0.6693	0.6283	0.8606	0.7941	0.9992	1.2351	0.8644
M07_50	M	1.4261	0.6626	0.5784	0.8563	0.7858	1.0189	1.2248	0.8545
M07_66	M	1.4075	0.6441	0.6166	0.8216	0.7491	0.9920	1.1989	0.8370
M07_73	M	1.4225	0.6525	0.6032	0.8459	0.7746	1.0169	1.2173	0.8517
M08_43	M	1.4318	0.6643	0.5448	0.8816	0.7965	1.0211	1.2371	0.8576
M08_47	M	1.4277	0.6673	0.6429	0.8653	0.7726	1.0030	1.2163	0.8612
M08_52	M	1.4329	0.6576	0.5922	0.8778	0.7851	1.0159	1.2327	0.8602
M08_57	M	1.4314	0.6466	0.6197	0.8583	0.7580	1.0243	1.2332	0.8567
M08_58	M	1.4392	0.6912	0.5788	0.8943	0.7928	1.0299	1.2429	0.8716
M08_61	M	1.4319	0.6640	0.5393	0.8697	0.7894	1.0279	1.2409	0.8552
M08_70	M	1.4195	0.6713	0.5286	0.8822	0.7686	1.0127	1.2311	0.8491
M08_72	M	1.4392	0.6710	0.6372	0.8757	0.7720	1.0451	1.2365	0.8729
M08_75	M	1.4156	0.6739	0.6063	0.8667	0.7694	0.9795	1.2073	0.8505
M08_77	M	1.4207	0.6726	0.6291	0.8824	0.7374	1.0069	1.2208	0.8582
M08_79	M	1.4406	0.6839	0.5876	0.8881	0.7958	1.0380	1.2421	0.8726
M13_11	M	1.4092	0.6525	0.5744	0.8589	0.7660	1.0031	1.2060	0.8435
M13_14	M	1.4072	0.6507	0.5628	0.8612	0.7681	0.9855	1.2047	0.8388
M13_15	M	1.4427	0.6922	0.6057	0.9119	0.7973	1.0141	1.2408	0.8770
M13_16	M	1.4268	0.6663	0.5064	0.8602	0.7815	1.0285	1.2421	0.8475
M13_20	M	1.4090	0.6593	0.5292	0.8707	0.7762	1.0181	1.2119	0.8442
M13_21	M	1.3980	0.6462	0.5454	0.8678	0.7618	0.9670	1.1963	0.8307
M13_22	M	1.4252	0.6881	0.5628	0.8980	0.7946	1.0318	1.2236	0.8665
M13_26	M	1.4223	0.6696	0.5752	0.8835	0.7871	1.0234	1.2190	0.8596

M13_28	M	1.4263	0.6610	0.5645	0.8583	0.7655	0.9962	1.2353	0.8468
M13_29	M	1.3999	0.6650	0.5094	0.8494	0.7671	0.9947	1.2050	0.8318
M13_31	M	1.4133	0.6497	0.6050	0.8234	0.7510	1.0045	1.2105	0.8407
M13_33	M	1.4075	0.6480	0.5550	0.8644	0.7559	0.9884	1.2113	0.8372
M13_34	M	1.3774	0.6262	0.5320	0.8047	0.7326	0.9531	1.1772	0.8043
M13_35	M	1.3996	0.6424	0.5265	0.8461	0.7602	0.9913	1.2035	0.8283
M13_49	M	1.4363	0.6859	0.6198	0.8917	0.7823	1.0151	1.2326	0.8712
M13_51	M	1.4098	0.6593	0.5622	0.8522	0.7647	1.0122	1.2102	0.8435
M13_54	M	1.4084	0.6733	0.5693	0.8589	0.7671	0.9955	1.2055	0.8449
M13_55	M	1.4466	0.6975	0.6284	0.8873	0.8075	1.0207	1.2379	0.8799
M13_56	M	1.4151	0.6514	0.5161	0.8507	0.7787	1.0035	1.2232	0.8373
M13_64	M	1.4359	0.6663	0.5756	0.8933	0.7975	1.0027	1.2367	0.8620
M13_68	M	1.4331	0.6726	0.5958	0.8859	0.7904	1.0162	1.2303	0.8652
M13_78	M	1.3864	0.6226	0.5573	0.8105	0.7458	0.9550	1.1809	0.8120
M13_8	M	1.4091	0.6381	0.4777	0.8354	0.7626	0.9900	1.2268	0.8218
M13_80	M	1.3887	0.6514	0.5469	0.8412	0.7532	0.9581	1.1843	0.8225

Table S2. RAD sequence data of parents and F2 individuals. Raw reads have been deposited at DNA Data Bank of Japan (DDBJ accession: DRA005547).

			DRA accession	
Individual ID	Sex	Generation	Experiment	Run
F07_2	female	F2	DRX081131	DRR087302
F07_37	female	F2	DRX081132	DRR087303
F07_45	female	F2	DRX081133	DRR087304
F07_60	female	F2	DRX081134	DRR087305
F07_65	female	F2	DRX081135	DRR087306
F07_69	female	F2	DRX081136	DRR087307
F07_9	female	F2	DRX081137	DRR087308
F08_41	female	F2	DRX081138	DRR087309
F08_42	female	F2	DRX081139	DRR087310
F08_44	female	F2	DRX081140	DRR087311
F08_59	female	F2	DRX081141	DRR087312
F08_62	female	F2	DRX081142	DRR087313
F08_63	female	F2	DRX081143	DRR087314
F08_71	female	F2	DRX081144	DRR087315
F08_74	female	F2	DRX081145	DRR087316
F08_76	female	F2	DRX081146	DRR087317
F13_1	female	F2	DRX081147	DRR087318
F13_12	female	F2	DRX081148	DRR087319
F13_13	female	F2	DRX081149	DRR087320
F13_17	female	F2	DRX081150	DRR087321
F13_18	female	F2	DRX081151	DRR087322
F13_19	female	F2	DRX081152	DRR087323
F13_23	female	F2	DRX081153	DRR087324
F13_24	female	F2	DRX081154	DRR087325
F13_3	female	F2	DRX081155	DRR087326
F13_30	female	F2	DRX081156	DRR087327
F13_32	female	F2	DRX081157	DRR087328
F13_36	female	F2	DRX081158	DRR087329
F13_38	female	F2	DRX081159	DRR087330
F13_39	female	F2	DRX081160	DRR087331
F13_4	female	F2	DRX081161	DRR087332
F13_40	female	F2	DRX081162	DRR087333
F13_46	female	F2	DRX081163	DRR087334
F13_48	female	F2	DRX081164	DRR087335
F13_5	female	F2	DRX081165	DRR087336
F13_53	female	F2	DRX081166	DRR087337
F13_6	female	F2	DRX081167	DRR087338

F13_67	female	F2	DRX081168	DRR087339
F13_7	female	F2	DRX081169	DRR087340
F13_81	female	F2	DRX081170	DRR087341
M07_10	male	F2	DRX081171	DRR087342
M07_27	male	F2	DRX081172	DRR087343
M07_50	male	F2	DRX081173	DRR087344
M07_66	male	F2	DRX081174	DRR087345
M07_73	male	F2	DRX081175	DRR087346
M08_43	male	F2	DRX081176	DRR087347
M08_47	male	F2	DRX081177	DRR087348
M08_52	male	F2	DRX081178	DRR087349
M08_57	male	F2	DRX081179	DRR087350
M08_58	male	F2	DRX081180	DRR087351
M08_61	male	F2	DRX081181	DRR087352
M08_70	male	F2	DRX081182	DRR087353
M08_72	male	F2	DRX081183	DRR087354
M08_75	male	F2	DRX081184	DRR087355
M08_77	male	F2	DRX081185	DRR087356
M08_79	male	F2	DRX081186	DRR087357
M13_11	male	F2	DRX081187	DRR087358
M13_14	male	F2	DRX081188	DRR087359
M13_15	male	F2	DRX081189	DRR087360
M13_16	male	F2	DRX081190	DRR087361
M13_20	male	F2	DRX081191	DRR087362
M13_21	male	F2	DRX081192	DRR087363
M13_22	male	F2	DRX081193	DRR087364
M13_26	male	F2	DRX081194	DRR087365
M13_28	male	F2	DRX081195	DRR087366
M13_29	male	F2	DRX081196	DRR087367
M13_31	male	F2	DRX081197	DRR087368
M13_33	male	F2	DRX081198	DRR087369
M13_34	male	F2	DRX081199	DRR087370
M13_35	male	F2	DRX081200	DRR087371
M13_49	male	F2	DRX081201	DRR087372
M13_51	male	F2	DRX081202	DRR087373
M13_54	male	F2	DRX081203	DRR087374
M13_55	male	F2	DRX081204	DRR087375
M13_56	male	F2	DRX081205	DRR087376
M13_64	male	F2	DRX081206	DRR087377
M13_68	male	F2	DRX081207	DRR087378
M13_78	male	F2	DRX081208	DRR087379

M13_8	male	F2	DRX081209	DRR087380
M13_80	male	F2	DRX081210	DRR087381
GF07	male	P	DRX081211	DRR087382
GF08	male	P	DRX081212	DRR087383
GF12	male	P	DRX081213	DRR087384
GF13	male	P	DRX081214	DRR087385
GM07	female	P	DRX081215	DRR087386
GM08	female	P	DRX081216	DRR087387
GM12	female	P	DRX081217	DRR087388
GM13	female	P	DRX081218	DRR087389

Table S3. Candidate genes and their GO terms in the scaffolds with QTL. *Drosophila melanogaster* was used for RefSeq protein ID.

								Category of gene functions for candidate gene search									
Linkage group	scaffold	Locus ID	start	stop	QTL region (or closest QTL)	RefSeq protein ID	Gene.Name	cell proliferation	cell size/cell growth	wing size	regulation of growth	insulin-like growth factor/insulin receptor signaling patway	Wnt signaling pathway	EGFR signaling pathway	EGF-like domain	Hippo signaling pathway	TOR signaling pathway
2	scaffold 350	evm.model.scaffold350_cov98.73 EVM	964778	1097768		NP_001096924	Megalin(mgl)					IPR009030:Insulin-like growth factor binding protein			IPR000742:Epidermal growth factor-like domain		
2	scaffold 350	evm.model.scaffold350_cov98.108 EVM	1524920	1531752		NP_524045	araucan(ara)				GO:0045926~negative regulation of growth						
2	scaffold 350	evm.model.scaffold350_cov98.111 EVM	1558555	1586270		NP_524047	mirror(mirr)				GO:0045926~negative regulation of growth						
2	scaffold 350	evm.model.scaffold350_cov98.149 EVM	2143088	2143711		NP_525062	CG10798 gene product from transcript CG10798-RB(Myc)	GO:0008283~cell proliferation	GO:0001558~regulation of cell growth;GO:0008361~regulation of cell size;0030307~positive regulation of cell growth;GO:0045793~positive regulation of cell size	GO:0044719~regulation of imaginal disc-derived wing size	GO:0045927~positive regulation of growth;GO:0048639~positive regulation of developmental growth		dme04310:Wnt signaling pathway			dme04391:Hippo signaling pathway	
2	scaffold 350	evm.model.scaffold350_cov98.186 EVM	2662042	2665126		NP_611856	CG2980 gene product from transcript CG2980-RA(thoc5)	GO:0008283~cell proliferation									
2	scaffold 350	evm.model.scaffold350_cov98.193 EVM	2796174	2797924		NP_001260391	crooked legs(crol)						GO:0030178~negative regulation of Wnt signaling pathway				
2	scaffold 350	evm.model.scaffold350_cov98.243 EVM	3348451	3349209		NP_610454	tsunagi(tsu)							GO:0007173~epidermal growth factor receptor signaling pathway			
2	scaffold 350	evm.model.scaffold350_cov98.254 EVM	3531820	3533379		NP_724096	Suppressor of cytokine signaling at 36E(Socs36E)							GO:0042059~negative regulation of epidermal growth factor receptor signaling pathway			
2	scaffold 350	evm.model.scaffold350_cov98.272 EVM	3657247	3662582		NP_477243	crooked legs(crol)						GO:0030178~negative regulation of Wnt signaling pathway				
2	scaffold 350	evm.model.scaffold350_cov98.275 EVM	3667013	3668326		NP_573019	CG12608 gene product from transcript CG12608-RA(CG12608)	GO:0008283~cell proliferation									
2	scaffold 350	evm.model.scaffold350_cov98.276 EVM	3668851	3672455		NP_001137978	CG8786 gene product from transcript CG8786-RG(CG8786)						GO:0016055~Wnt signaling pathway				
2	scaffold 350	evm.model.scaffold350_cov98.300 EVM	3972850	3973336		NP_524129	Keren(Krn)	0008284~positive regulation of cell proliferation						GO:0007173~epidermal growth factor receptor signaling pathway;GO:0042058~regulation of epidermal growth factor receptor signaling pathway,GO:0045741~positive regulation of epidermal growth factor-activated receptor activity	IPR000742:Epidermal growth factor-like domain	dme04391:Hippo signaling pathway	

2	scaffold 350	evm.model.scaffold350_cov98.325 EVM	4228695	4231856	HW	NP_001260030	dumpy(dpy)			GO:0044719~regulation of imaginal disc-derived wing size		IPR009030:Insulin-like growth factor binding protein			IPR000742:Epidermal growth factor-like domain		
5	scaffold 119	evm.model.scaffold119_cov99.28 EVM	362293	427870	TW, GM	NP_001188674	CG11940 gene product from transcript CG11940-RB(pico)		GO:0008361~regulation of cell size		GO:0048639~positive regulation of developmental growth						
5	scaffold 119	evm.model.scaffold119_cov99.35 EVM	533183	537067	TW	NP_649531	humpty dumpty(hd)	GO:0008283~cell proliferation									
5	scaffold 119	evm.model.scaffold119_cov99.41 EVM	560402	561813	TW	NP_649051	Charged multivesicular body protein 1(Chmp1)							GO:0042059~negative regulation of epidermal growth factor receptor signaling pathway			
5	scaffold 119	evm.model.scaffold119_cov99.53 EVM	741244	743446	TW	NP_611691	CG4207 gene product from transcript CG4207-RA(bonsai)				GO:0040008~regulation of growth						
5	scaffold 119	evm.model.scaffold119_cov99.83 EVM	1186599	1191388	TW	NP_523946	sulfateless(sfl)						GO:0060828~regulation of canonical Wnt signaling pathway				
5	scaffold 119	evm.model.scaffold119_cov99.229 EVM	2471757	2478756	TW	NP_996243	Signal-transducer and activator of transcription protein at 92E(Stat92E)	GO:0008284~positive regulation of cell proliferation					GO:0030178~negative regulation of Wnt signaling pathway				
5	scaffold 119	evm.model.scaffold119_cov99.236 EVM	2543793	2550978		NP_724070	Cadherin-N(CadN)					,IPR009030:Insulin-like growth factor binding protein, N-terminal			IPR000742:Epidermal growth factor-like domain		
5	scaffold 119	evm.model.scaffold119_cov99.237 EVM	2551641	2556023		NP_724069	Cadherin-N(CadN)					,IPR009030:Insulin-like growth factor binding protein, N-terminal			IPR000742:Epidermal growth factor-like domain		
5	scaffold 119	evm.model.scaffold119_cov99.248 EVM	2711234	2717267		NP_724069	Cadherin-N(CadN)					,IPR009030:Insulin-like growth factor binding protein, N-terminal			IPR000742:Epidermal growth factor-like domain		
5	scaffold 119	evm.model.scaffold119_cov99.250 EVM	2791531	2809730		NP_724071	Cadherin-N(CadN)					,IPR009030:Insulin-like growth factor binding protein, N-terminal			IPR000742:Epidermal growth factor-like domain		
5	scaffold 119	evm.model.scaffold119_cov99.274 EVM	2943269	2947825		NP_651349	Cadherin 96Ca(Cad96Ca)		GO:0045792~negative regulation of cell size								
5	scaffold 119	evm.model.scaffold119_cov99.283 EVM	2996937	3011314		NP_523723	vestigial(vg)	GO:0042127~regulation of cell proliferation		GO:0044719~regulation of imaginal disc-derived wing size							
5	scaffold 119	evm.model.scaffold119_cov99.292 EVM	3068329	3070185		NP_523983	division abnormally delayed(dally)				GO:0040014~regulation of multicellular organism growth,GO:0045570~regulation of imaginal disc growth		GO:0016055~Wnt signaling pathway			dme04391:Hippo signaling pathway	
5	scaffold 119	evm.model.scaffold119_cov99.293 EVM	3079438	3080991		NP_523983	division abnormally delayed(dally)				GO:0040014~regulation of multicellular organism growth,GO:0045570~regulation of imaginal disc growth		GO:0016055~Wnt signaling pathway			dme04391:Hippo signaling pathway	
5	scaffold 119	evm.model.scaffold119_cov99.300 EVM	3118683	3120895		NP_996075	dally-like(dlp)				GO:0045570~regulation of imaginal disc growth		GO:0016055~Wnt signaling pathway,GO:0030177~positive regulation of Wnt signaling pathway,GO:0030178~negative regulation of Wnt signaling pathway				

5	scaffold 119	evm.model.scaffold119_cov99.304 EVM	3135234	3136961		NP_476976	dreadlocks(dock)					GO:0008286~insulin receptor signaling pathway,GO:0046627~negative regulation of insulin receptor signaling pathway					
5	scaffold 119	evm.model.scaffold119_cov99.389 EVM	3931062	3933029		NP_573382	kekkon5(kek5)							GO:0042059~negative regulation of epidermal growth factor receptor signaling pathway			
5	scaffold 119	evm.model.scaffold119_cov99.445 EVM	4734777	4746771		NP_001188748	taiman(tai)				GO:0045927~positive regulation of growth						
5	scaffold 119	evm.model.scaffold119_cov99.462 EVM	5058213	5059355		NP_524124	target of Poxn(tap)						GO:2000095~regulation of Wnt signaling pathway				
5	scaffold 119	evm.model.scaffold119_cov99.489 EVM	5310123	5311332		NP_001260034	dumpy(dpy)			GO:0044719~regulation of imaginal disc-derived wing size,		IPR009030:Insulin-like growth factor binding protein			IPR000742:Epidermal growth factor-like domain		
5	scaffold 119	evm.model.scaffold119_cov99.567 EVM	6258243	6285759		NP_524812	frizzled(fz)						GO:0016055~Wnt signaling pathway				
5	scaffold 119	evm.model.scaffold119_cov99.584 EVM	6378979	6394147		NP_477243	crooked legs(crol)						GO:0030178~negative regulation of Wnt signaling pathway				
5	scaffold 119	evm.model.scaffold119_cov99.586 EVM	6397832	6398943		NP_650048	Translationally controlled tumor protein ortholog (H. sapiens)(Tctp)		GO:0045793~positive regulation of cell size								
5	scaffold 50	evm.model.scaffold50_cov100.13 EVM	242793	243686	BL, TW	NP_476972	Wnt oncogene analog 4(Wnt4)						GO:0016055~Wnt signaling pathway				
5	scaffold 50	evm.model.scaffold50_cov100.14 EVM	261312	285637	BL, TW	NP_523502	wingless(wg)	GO:0008284~positive regulation of cell proliferation;GO:0042127~regulation of cell proliferation		GO:0044719~regulation of imaginal disc-derived wing size			GO:0016055~Wnt signaling pathway;GO:0060070~canonical Wnt signaling pathway			dme04391:Hippo signaling pathway	
5	scaffold 50	evm.model.scaffold50_cov100.15 EVM	298513	304172	BL, TW	NP_609108	Wnt oncogene analog 6(Wnt6)			GO:0044719~regulation of imaginal disc-derived wing size			GO:0016055~Wnt signaling pathway				
5	scaffold 50	evm.model.scaffold50_cov100.18 EVM	326261	343685	BL, TW	NP_609109	Wnt oncogene analog 10(Wnt10)						GO:0016055~Wnt signaling pathway				
5	scaffold 50	evm.model.scaffold50_cov100.32 EVM	459230	462142	BL, TW	NP_723814	CG9293 gene product from transcript CG9293-RB(CG9293)	GO:0008285~negative regulation of cell proliferation									
5	scaffold 50	evm.model.scaffold50_cov100.36 EVM	487463	489203	BL, TW	NP_523625	Actin 42A(Act42A)									dme04391:Hippo signaling pathway	
5	scaffold 50	evm.model.scaffold50_cov100.40 EVM	507945	513731	BL, TW	NP_001260390	crooked legs(crol)						GO:0030178~negative regulation of Wnt signaling pathway				
5	scaffold 50	evm.model.scaffold50_cov100.65 EVM	852836	855018		NP_569915	Nucleostemin ortholog (H. sapiens) 3(Ns3)				GO:0040018~positive regulation of multicellular organism growth	O:0046626~regulation of insulin receptor signaling pathway,GO:0051168~nuclear export					
5	scaffold 50	evm.model.scaffold50_cov100.72 EVM	868077	871247		NP_648927	sina homologue(sinah)						dme04310:Wnt signaling pathway				
5	scaffold 50	evm.model.scaffold50_cov100.77 EVM	896745	905689		NP_001188806	wing blister(wb)								IPR000742:Epidermal growth factor-like domain		
5	scaffold 50	evm.model.scaffold50_cov100.79 EVM	928822	946370		NP_476790	wing blister(wb)								IPR000742:Epidermal growth factor-like domain		

5	scaffold 50	evm.model.scaffold50_cov100.82 EVM	965811	971740		NP_723870	wing blister(wb)								IPR000742:Epidermal growth factor-like domain		
5	scaffold 51	evm.model.scaffold51_cov93.5 EVM	18112	46317	BL, TW	NP_001014565	vein(vn)	GO:0008284~positive regulation of cell proliferation						GO:0045742~positive regulation of epidermal growth factor receptor signaling pathway;GO:0005154~epidermal growth factor receptor binding	IPR000742:Epidermal growth factor-like domain	dme04391:Hippo signaling pathway	
5	scaffold 51	evm.model.scaffold51_cov93.32 EVM	484779	491457	BL, TW, EL	NP_001245496	terribly reduced optic lobes(trol)								IPR000742:Epidermal growth factor-like domain		
5	scaffold 51	evm.model.scaffold51_cov93.35 EVM	524898	558050	BL, GW, TW, EL	NP_001245496	terribly reduced optic lobes(trol)								IPR000742:Epidermal growth factor-like domain		
5	scaffold 51	evm.model.scaffold51_cov93.44 EVM	808934	811022	BL, GW, TW, EL	NP_477245	crooked legs(crol)						GO:0030178~negative regulation of Wnt signaling pathway				
5	scaffold 51	evm.model.scaffold51_cov93.45 EVM	822907	825658	BL, GW, TW, EL	NP_001260390	crooked legs(crol)						GO:0030178~negative regulation of Wnt signaling pathway				
5	scaffold 51	evm.model.scaffold51_cov93.63 EVM	994027	998306		NP_001137938	approximated(app)									dme04391:Hippo signaling pathway	
5	scaffold 52	evm.model.scaffold52_cov104.26 EVM	426984	429521	BL, GW, TW, EL	NP_524437	E2F transcription factor 1(E2f1)	GO:0008284~positive regulation of cell proliferation									
5	scaffold 52	evm.model.scaffold52_cov104.27 EVM	435892	439392	BL, GW, TW, EL	NP_788604	pumilio(pum)							GO:0042059~negative regulation of epidermal growth factor receptor signaling pathway			
5	scaffold 52	evm.model.scaffold52_cov104.30 EVM	464869	473330	BL, GW, TW, EL	NP_001262404	pumilio(pum)							GO:0042059~negative regulation of epidermal growth factor receptor signaling pathway			
5	scaffold 52	evm.model.scaffold52_cov104.32 EVM	500082	502887	BL, GW, TW, EL	NP_731316	pumilio(pum)							GO:0042059~negative regulation of epidermal growth factor receptor signaling pathway			
5	scaffold 52	evm.model.scaffold52_cov104.44 EVM	606806	608367	BL, GW, TW, EL	NP_477329	CG4063 gene product from transcript CG4063-RA(ebi)						dme04310:Wnt signaling pathway	GO:0007173~epidermal growth factor receptor signaling pathway,GO:0042058~regulation of epidermal growth factor receptor signaling pathway			
5	scaffold 52	evm.model.scaffold52_cov104.47 EVM	668346	670827	BL, GW, TW, EL	NP_524726	Ribosomal protein L8(RpL8)		GO:0008361~regulation of cell size			GO:0008286~insulin receptor signaling pathway					
5	scaffold 52	evm.model.scaffold52_cov104.58 EVM	760279	764825	BL, GW, TW, EL	NP_523790	brother of tout-velu(botv)						GO:0016055~Wnt signaling pathway				
5	scaffold 52	evm.model.scaffold52_cov104.78 EVM	925418	940740		NP_477130	corkscrew(csw)							GO:0007173~epidermal growth factor receptor signaling pathway			
5	scaffold 52	evm.model.scaffold52_cov104.94 EVM	1022549	1024801		NP_523451	asteroid(ast)							GO:0007173~epidermal growth factor receptor signaling pathway			
5	scaffold 52	evm.model.scaffold52_cov104.133 EVM	1243014	1250081		NP_001285267	Topoisomerase 1(Top1)	GO:0008283~cell proliferation									
5	scaffold 52	evm.model.scaffold52_cov104.170 EVM	1645431	1652570		NP_001259924	eyes shut(eys)								IPR000742:Epidermal growth factor-like domain		

5	scaffold 52	evm.model.scaffold52_cov104.185 EVM	1765171	1771975		NP_729306	pebble(pbl)		GO:0045792~negative regulation of cell size				GO:0090090~negative regulation of canonical Wnt signaling pathway				
5	scaffold 52	evm.model.scaffold52_cov104.196 EVM	1868428	1871069		NP_649850	Ras-related GTP binding A/B ortholog (H. sapiens)(RagA-B)		GO:0030307~positive regulation of cell growth								GO:0032008~positive regulation of TOR signaling
5	scaffold 52	evm.model.scaffold52_cov104.202 EVM	1884037	1885511		NP_524409	Heparan sulfate 6-O-sulfotransferase(Hs6st)						GO:0060828~regulation of canonical Wnt signaling pathway				
5	scaffold 52	evm.model.scaffold52_cov104.206 EVM	1897893	1902579		NP_572930	Ajuba LIM protein(jub)									GO:0035329~hippo signaling,GO:0035331~negative regulation of hippo signaling	
5	scaffold 52	evm.model.scaffold52_cov104.216 EVM	1932385	1952631		NP_524987	Sulfated(Sulf1)						GO:0030178~negative regulation of Wnt signaling pathway	GO:0042059~negative regulation of epidermal growth factor receptor signaling pathway			
5	scaffold 52	evm.model.scaffold52_cov104.280 EVM	2394439	2399680		NP_728307	CG11940 gene product from transcript CG11940-RB(pico)		GO:0008361~regulation of cell size		GO:0048639~positive regulation of developmental growth						
5	scaffold 52	evm.model.scaffold52_cov104.282 EVM	2420123	2445488		NP_001245768	vielfaltig(vfl)			GO:0044719~regulation of imaginal disc-derived wing size							
5	scaffold 52	evm.model.scaffold52_cov104.286 EVM	2466428	2469269		NP_477087	Activated Cdc42 kinase-like(Ack-like)	GO:0042127~regulation of cell proliferatio									
5	scaffold 52	evm.model.scaffold52_cov104.288 EVM	2471617	2473853		NP_477086	Activated Cdc42 kinase-like(Ack-like)	GO:0042127~regulation of cell proliferation									
6	scaffold 216	evm.model.scaffold216_cov103.3 EVM	17386	19753	BL, GM, EL	NP_523498	Menin 1(Mnn1)	GO:0008285~negative regulation of cell proliferation									
6	scaffold 224	evm.model.scaffold224_cov105.2 EVM	28687	30753		NP_523559	kekkon-1(kek1)							GO:0042059~negative regulation of epidermal growth factor receptor signaling pathway			
6	scaffold 224	evm.model.scaffold224_cov105.13 EVM	158820	168788		NP_652731	Desaturase 1(Desat1)		GO:0045793~positive regulation of cell size								
6	scaffold 224	evm.model.scaffold224_cov105.21 EVM	246334	248552		NP_523551	kekkon-2(kek2)							GO:0042059~negative regulation of epidermal growth factor receptor signaling pathway			
6	scaffold 224	evm.model.scaffold224_cov105.34 EVM	461993	466436		NP_572988	pudgy(pdgy)					GO:0046627~negative regulation of insulin receptor signaling pathway					
6	scaffold 224	evm.model.scaffold224_cov105.35 EVM	467347	470069		NP_572988	pudgy(pdgy)					GO:0046627~negative regulation of insulin receptor signaling pathway					
6	scaffold 224	evm.model.scaffold224_cov105.64 EVM	675242	682045	EL	NP_572988	pudgy(pdgy)					GO:0046627~negative regulation of insulin receptor signaling pathway					
6	scaffold 224	evm.model.scaffold224_cov105.66 EVM	684479	684849		NP_572988	pudgy(pdgy)					GO:0046627~negative regulation of insulin receptor signaling pathway					
6	scaffold 224	evm.model.scaffold224_cov105.100 EVM	1050689	1052309		NP_477311	decapentaplegic(dpp)	GO:0008285~negative regulation of cell proliferation;GO:0042127~regulation of cell proliferation									

6	scaffold 224	evm.model.scaffold224_cov105.110 EVM	1099063	1099531		NP_524741	Calcineurin B(CanB)						dme04310:Wnt signaling pathway				
6	scaffold 224	evm.model.scaffold224_cov105.113 EVM	1130323	1135449		NP_477311	decapentaplegic(dpp)	GO:0008285~negative regulation of cell proliferation;GO:0042127~regulation of cell proliferation									
6	scaffold 224	evm.model.scaffold224_cov105.154 EVM	1550194	1554468		NP_476614	CG4162 gene product from transcript CG4162-RA(lace)				GO:0040008~regulation of growth		GO:0030111~regulation of Wnt signaling pathway				
6	scaffold 588	evm.model.scaffold588_cov103.27 EVM	207016	211810	GM	NP_610216	Brahma associated protein 170kD(Bap170)							GO:0042059~negative regulation of epidermal growth factor receptor signaling pathway			
6	scaffold 588	evm.model.scaffold588_cov103.29 EVM	216377	224958		NP_599132	Syntaxin 5(Syx5)	GO:0008283~cell proliferation									
17	scaffold 54	evm.model.scaffold54_cov100.7 EVM	73663	103294		NP_524672	tribbles(trbl)					GO:0046627~negative regulation of insulin receptor signaling pathway					
17	scaffold 54	evm.model.scaffold54_cov100.26 EVM	265287	269159		NP_476726	small wing(sl)		GO:0030307~positive regulation of cell growth					GO:0042059~negative regulation of epidermal growth factor receptor signaling pathway			
17	scaffold 54	evm.model.scaffold54_cov100.36 EVM	349828	382000	GM	NP_523705	off-track(otk)						GO:0090090~negative regulation of canonical Wnt signaling pathway				
22	scaffold 59	evm.model.scaffold59_cov101.2 EVM	49321	51497		NP_609615	kekkon4(kek4)						GO:0042059~negative regulation of epidermal growth factor receptor signaling pathway				
22	scaffold 59	evm.model.scaffold59_cov101.7 EVM	97465	105629		NP_723540	CG5686 gene product from transcript CG5686-RB(chico)		GO:0008361~regulation of cell size;GO:0030307~positive regulation of cell growth;GO:0045793~positive regulation of cell size		GO:0035264~multicellular organism growth,GO:0040014~regulation of multicellular organism growth	GO:0008286~insulin receptor signaling pathway;GO:0048009~insulin-like growth factor receptor signaling pathway					
22	scaffold 59	evm.model.scaffold59_cov101.9 EVM	123761	129339		NP_001287034	charybde(chrb)				GO:0045926~negative regulation of growth						GO:0032006~regulation of TOR signaling
22	scaffold 59	evm.model.scaffold59_cov101.29 EVM	314157	335314		NP_001245407	CG33978 gene product from transcript CG33978-RD(CG33978)								IPR000742:Epidermal growth factor-like domain		
22	scaffold 59	evm.model.scaffold59_cov101.38 EVM	488710	489579		NP_476725	seven in absentia(sina)							dme04310:Wnt signaling pathway			
22	scaffold 59	evm.model.scaffold59_cov101.44 EVM	573178	574154		NP_651887	CG2245 gene product from transcript CG2245-RA(nero)	GO:0008283~cell proliferation	GO:0016049~cell growth								
22	scaffold 59	evm.model.scaffold59_cov101.47 EVM	577854	578531		NP_647948	p53-related protein kinase ortholog(Prpk)		GO:0030307~positive regulation of cell growth		GO:0040018~positive regulation of multicellular organism growth						GO:0032008~positive regulation of TOR signaling,
22	scaffold 59	evm.model.scaffold59_cov101.90 EVM	1139083	1141903	HL	NP_722830	Hepatocyte growth factor regulated tyrosine kinase substrate(Hrs)						GO:0042058~regulation of epidermal growth factor receptor signaling pathway,GO:0042059~negative regulation of epidermal growth factor receptor signaling pathway				
22	scaffold 59	evm.model.scaffold59_cov101.94 EVM	1188837	1195321	HL	NP_523779	SMAD specific E3 ubiquitin protein ligase(Smurf)				GO:0040008~regulation of growth						

22	scaffold 59	evm.model.scaffold59_cov101.117 EVM	1413588	1424101	HL	NP_524155	frizzled 2(fz2)							GO:0016055~Wnt signaling pathway			
22	scaffold 59	evm.model.scaffold59_cov101.120 EVM	1431745	1435762		NP_476708	RNA polymerase I 135kD subunit(RpI135)		GO:0016049~cell growth,GO:0045793~positive regulation of cell size								
22	scaffold 59	evm.model.scaffold59_cov101.122 EVM	1441196	1459710		NP_001259803	split ends(spen)						GO:0007173~epidermal growth factor receptor signaling pathway	GO:0016055~Wnt signaling pathway,GO:0030177~positive regulation of Wnt signaling pathway			
22	scaffold 59	evm.model.scaffold59_cov101.127 EVM	1553691	1555025		NP_477295	CG8846 gene product from transcript CG8846-RA(Thor)		GO:0001558~regulation of cell growth;GO:0045792~negative regulation of cell size,								
22	scaffold 59	evm.model.scaffold59_cov101.144 EVM	1810536	1817119		NP_001097582	CG32082 gene product from transcript CG32082-RA(CG32082)					GO:0008286~insulin receptor signaling pathway					
22	scaffold 59	evm.model.scaffold59_cov101.155 EVM	1955335	1960510		NP_001096855	Rho GTPase activating protein at 1A(RhoGAP1A)		GO:0045792~negative regulation of cell size								
22	scaffold 59	evm.model.scaffold59_cov101.182 EVM	2293153	2295652		NP_648927	sina homologue(sinah)							dme04310:Wnt signaling pathway			
22	scaffold 59	evm.model.scaffold59_cov101.196 EVM	2427154	2437467		NP_723541	basket(bsk)							GO:0016055~Wnt signaling pathway		Hippo signaling pathway	
22	scaffold 59	evm.model.scaffold59_cov101.199 EVM	2494696	2496185		NP_477424	Phosphatase and tensin homolog(Pten)	GO:0008283~cell proliferation,GO:0008285~negative regulation of cell proliferation	GO:0008361~regulation of cell size;GO:0045792~negative regulation of cell size		GO:0040014~regulation of multicellular organism growth,GO:0040015~negative regulation of multicellular organism growth	GO:0008286~insulin receptor signaling pathway;GO:0046627~negative regulation of insulin receptor signaling pathway					
22	scaffold 59	evm.model.scaffold59_cov101.219 EVM	2758996	2759514		NP_724946	longitudinals lacking(lola)			GO:0044719~regulation of imaginal disc-derived wing size							
22	scaffold 59	evm.model.scaffold59_cov101.221 EVM	2774258	2782358		NP_477243	crooked legs(crol)							GO:0030178~negative regulation of Wnt signaling pathway			
22	scaffold 59	evm.model.scaffold59_cov101.222 EVM	2801125	2802356		NP_788320	longitudinals lacking(lola)			GO:0044719~regulation of imaginal disc-derived wing size							
22	scaffold 59	evm.model.scaffold59_cov101.261 EVM	3191848	3194889		NP_524020	haywire(hay)	GO:0042127~regulation of cell proliferation			GO:0040008~regulation of growth						
X	scaffold 341	evm.model.scaffold341_cov52.31 EVM	337899	338449		NP_612045	CG3402 gene product from transcript CG3402-RA(CG3402)						GO:0030111~regulation of Wnt signaling pathway				
X	scaffold 341	evm.model.scaffold341_cov52.40 EVM	437841	440152		NP_001036454	conundrum(conu)	0008284~positive regulation of cell proliferation									
X	scaffold 341	evm.model.scaffold341_cov52.59 EVM	578706	584012	EW	NP_524392	CG7467 gene product from transcript CG7467-RE(osa)						GO:0016055~Wnt signaling pathway	GO:0042058~regulation of epidermal growth factor receptor signaling pathway			
X	scaffold 341	evm.model.scaffold341_cov52.60 EVM	585710	596078	HL	NP_996228	CG7467 gene product from transcript CG7467-RE(osa)						GO:0016055~Wnt signaling pathway	GO:0042058~regulation of epidermal growth factor receptor signaling pathway			
X	scaffold 341	evm.model.scaffold341_cov52.66 EVM	676019	678849		NP_524683	SHC-adaptor protein(Shc)							GO:0007173~epidermal growth factor receptor signaling pathwa			

X	scaffold 341	evm.model.scaffold341_cov52.74 EVM	779941	789292		NP_476618	CG7123 gene product from transcript CG7123-RA(LanB1)								IPR000742:Epidermal growth factor-like domain		
X	scaffold 644	evm.model.scaffold644_cov53.2 EVM	2869	6143		NP_648489	RIO kinase 1(RIOK1)				GO:0040018~positive regulation of multicellular organism growth						
X	scaffold 644	evm.model.scaffold644_cov53.9 EVM	30375	35177		NP_524812	frizzled(fz)						GO:0016055~Wnt signaling pathway				
X	scaffold 644	evm.model.scaffold644_cov53.15 EVM	67125	71120		NP_996208	Kinesin family member 19A ortholog(Kif19A)					GO:0008286~insulin receptor signaling pathway					
X	scaffold 644	evm.model.scaffold644_cov53.16 EVM	74637	83104		NP_524343	CG9829 gene product from transcript CG9829-RB(poly)					GO:0008286~insulin receptor signaling pathway					
X	scaffold 644	evm.model.scaffold644_cov53.53 EVM	310075	312143		NP_524177	gigas(gig)	GO:0008285~negative regulation of cell proliferation	GO:0045792~negative regulation of cell size			GO:0046627~negative regulation of insulin receptor signaling pathway	GO:0030178~negative regulation of Wnt signaling pathway				
X	scaffold 644	evm.model.scaffold644_cov53.58 EVM	378231	384398		NP_001188729	dachs(d)				GO:0045927~positive regulation of growth; GO:0045572~positive regulation of imaginal disc growth					dme04391:Hippo signaling pathway	
X	scaffold 644	evm.model.scaffold644_cov53.81 EVM	592402	596492	EW	NP_612113	cueball(cue)								IPR000742:Epidermal growth factor-like domain		
X	scaffold 644	evm.model.scaffold644_cov53.85 EVM	647197	656310		NP_523876	extra macrochaetae(emc)	GO:0008283~cell proliferation									
X	scaffold 644	evm.model.scaffold644_cov53.94 EVM	753850	754691		NP_610218	CG3183 gene product from transcript CG3183-RA(geminin)							GO:0042059~negative regulation of epidermal growth factor receptor signaling pathway			
